# Weighted gene co-expression network analysis identified hub genes critical to fatty acid composition in Gushi chicken breast muscle

**DOI:** 10.1186/s12864-023-09685-8

**Published:** 2023-10-07

**Authors:** Bin Zhai, Yinli Zhao, Hongtai Li, Shuaihao Li, Jinxing Gu, Hongyuan Zhang, Yanhua Zhang, Hong Li, Yadong Tian, Guoxi Li, Yongcai Wang

**Affiliations:** 1 College of Animal Science and Technology, Henan Agricultural University, Zhengzhou, 450046 China; 2 College of Biological Engineering, Henan University of Technology, Zheng Zhou, Henan Province 450001 People’s Republic of China; 3 Henan Key Laboratory for Innovation and Utilization of Chicken Germplasm Resources, Zhengzhou, 450046 P. R. China; 4 The Shennong Laboratory, Zhengzhou, 450046 China

**Keywords:** Chicken, Breast muscles, Fatty acid composition, WGCNA, Hub gene

## Abstract

**Background:**

The composition and content of fatty acids in the breast muscle are important factors influencing meat quality. In this study, we investigated the fatty acid composition and content in the breast muscle of Gushi chickens at different developmental stages (14 weeks, 22 weeks, and 30 weeks). Additionally, we utilized transcriptomic data from the same tissue and employed WGCNA and module identification methods to identify key genes associated with the fatty acid composition in Gushi chicken breast muscle and elucidate their regulatory networks.

**Results:**

Among them, six modules (blue, brown, green, light yellow, purple, and red modules) showed significant correlations with fatty acid content and metabolic characteristics. Enrichment analysis revealed that these modules were involved in multiple signaling pathways related to fatty acid metabolism, including fatty acid metabolism, PPAR signaling pathway, and fatty acid biosynthesis. Through analysis of key genes, we identified 136 genes significantly associated with fatty acid phenotypic traits. Protein–protein interaction network analysis revealed that nine of these genes were closely related to fatty acid metabolism. Additionally, through correlation analysis of transcriptome data, we identified 51 key ceRNA regulatory networks, including six central genes, 7 miRNAs, and 28 lncRNAs.

**Conclusion:**

This study successfully identified key genes closely associated with the fatty acid composition in Gushi chicken breast muscle, as well as their post-transcriptional regulatory networks. These findings provide new insights into the molecular regulatory mechanisms underlying the flavor characteristics of chicken meat and the composition of fatty acids in the breast muscle.

**Supplementary Information:**

The online version contains supplementary material available at 10.1186/s12864-023-09685-8.

## Background

With the improvement of living standards, people are paying increasing attention to the taste and nutritional quality of meat. The breast muscle of chicken is highly favored due to its rich content of fatty acids, which directly influence the taste, flavor, tenderness, and nutritional value of chicken meat [1]. However, the composition and content of these fatty acids are regulated by various factors such as genetics and nutrition [2–5]. Therefore, it is of great significance to understand the molecular regulatory mechanisms behind the fatty acid composition and content in chicken breast muscle. This understanding can provide guidance for developing nutritional strategies or conducting genetic improvements, thereby optimizing the taste, flavor, and nutritional value of chicken breast muscle. However, the molecular regulatory mechanisms underlying these characteristics are currently not well understood.

As a renowned local chicken breed in China, Gushi chicken is highly acclaimed for its delicious taste, unique flavor, and nutritional richness. It is well known that the texture and tenderness of chicken breast are closely associated with the content and composition of its fatty acids. Abundant fat content gives the meat a richer taste and increased tenderness, resulting in juicier meat [6, 7]. Additionally, the presence of unsaturated fatty acids improves the flexibility of muscle fibers [7]. The nutritional value of different fatty acids is also a key factor influencing meat quality. According to nutritional standards, increasing the intake of polyunsaturated fatty acids (PUFAs) can reduce the risk of cardiovascular diseases, certain cancers, asthma, and diabetes [8–10]. Therefore, maintaining a balance between unsaturated and saturated fatty acids and reducing the intake of saturated fats is an effective strategy for lowering the risk of these diseases [11]. In comparison to other meat products, chicken meat is rich in higher levels of unsaturated fatty acids and lower levels of saturated fats, conversely, pork and ruminant meats have higher levels of saturated fats and lower levels of polyunsaturated fatty acids [12–14]. Therefore, further study on the composition and content of chicken breast muscle fat is of great significance to meat quality and healthy diet.

Previous studies on the relationship between fatty acids and meat quality have primarily focused on the field of nutrition, often describing the composition and content of fatty acids in specific meat types without considering their connection to gene regulation. However, the application of transcriptome sequencing technology and Weighted Gene Co-expression Network Analysis (WGCNA) offers a new approach to explore the relationship between phenotype and gene expression profiles [15]. For example, WGCNA analysis of beef fatty acids has identified insulin and MAPK signaling pathways as central pathways associated with fatty acids, and ACTA1 and ALDOA as potential regulators of fatty acid synthesis [16]. In the poultry field, Gai et al. performed a combined analysis of transcriptome and metabolome using WGCNA in Beijing-You chickens and found that CBS, GATM, GAD6, PNPLA1, ItaE, and AMPD1 were associated with the accumulation of flavor components [17]. Li et al. conducted WGCNA analysis on fat content and six fatty acids in Gushi chickens, identifying genes related to fatty acid metabolism, phospholipid metabolism, and cholesterol metabolism [18]. Furthermore, the study found that non-coding RNAs are also involved in the regulation of biological processes related to intramuscular fat deposition [19]. For instance, gga-miR-140-5p promotes chicken intramuscular adipocyte differentiation by targeting RXRG, while gga-miR-18b-3p acts as an inhibitor of intramuscular adipocyte differentiation by targeting ACOT13 [20, 21]. Currently, research on lncRNAs and their competing endogenous RNA (ceRNA) regulatory networks related to fatty acids is relatively limited. One example of ceRNA regulation is the lncRNA MFNCR, which stimulates the differentiation of intramuscular adipocytes in chickens by sequestering miR-128-3p and miR-27b-3p [22]. Therefore, due to the limitations of previous fatty acid determination methods and transcriptome technologies, the composition, content, and molecular regulatory mechanisms of fatty acids in Gushi chicken breast muscle remain unclear.

In the Gushi chicken production process, the chickens are at different developmental stages: 14 weeks for muscle development, 22 weeks for adipocyte development, and 30 weeks for the peak of egg production. Therefore, in this study, we utilized three different developmental stages of breast muscle tissue from the Gushi chicken to conduct the following investigations: (1) Accurately constructing the fatty acid profile in the breast muscle of the Gushi chicken to understand the variations in fatty acid composition and content across different stages. (2) Utilizing WGCNA to identify potential genes and signaling pathways that influence the composition and content of fatty acids in the pectoralis muscle, revealing key genes involved in the formation of fatty acid meat quality traits. (3) Establishing a post-transcriptional ceRNA regulatory network for fatty acids in the pectoralis muscle of the Gushi chicken to investigate the molecular regulatory network governing the meat quality traits of fatty acids at the transcriptional level. These research findings contribute to a deeper understanding of the nutritional characteristics and meat quality formation mechanisms in chicken, providing scientific evidence and guidance for chicken production and meat quality improvement.

## Results

### Phenotypic data associated with FA composition

Through GC–MS analysis, a total of 49 fatty acids were detected in the breast muscle tissue of Guishi chickens at three different developmental stages. Additionally, 16 metabolic characteristics of fatty acids were calculated (Table S1). Among them, there were 14 saturated fatty acids (SFA), 21 monounsaturated fatty acids (MUFA), and 14 polyunsaturated fatty acids (PUFA). SFA, MUFA, and PUFA accounted for approximately 51.95% to 64.63%, 23.70% to 34.80%, and 11.66% to 13.26% of the total fatty acid content, respectively. Furthermore, eight dominant fatty acids were identified in the breast muscle of Gushi chickens. These included two saturated fatty acids, namely palmitic acid (C16:0) and stearic acid (C18:0), as well as six unsaturated fatty acids, including vaccenic acid (C18:1N12), oleic acid (C18:1N9C), arachidonic acid (C20:4N6), palmitoleic acid (C14:1T), and linoleic acid (C18:2N6). These data will be utilized for the correlation analysis between gene co-expression modules and phenotypic traits, contributing to the further investigation of the relationship between genes and fatty acid phenotypic characteristics.

### Weighted gene co-expression network construction and module identification

**Fig. 1:**
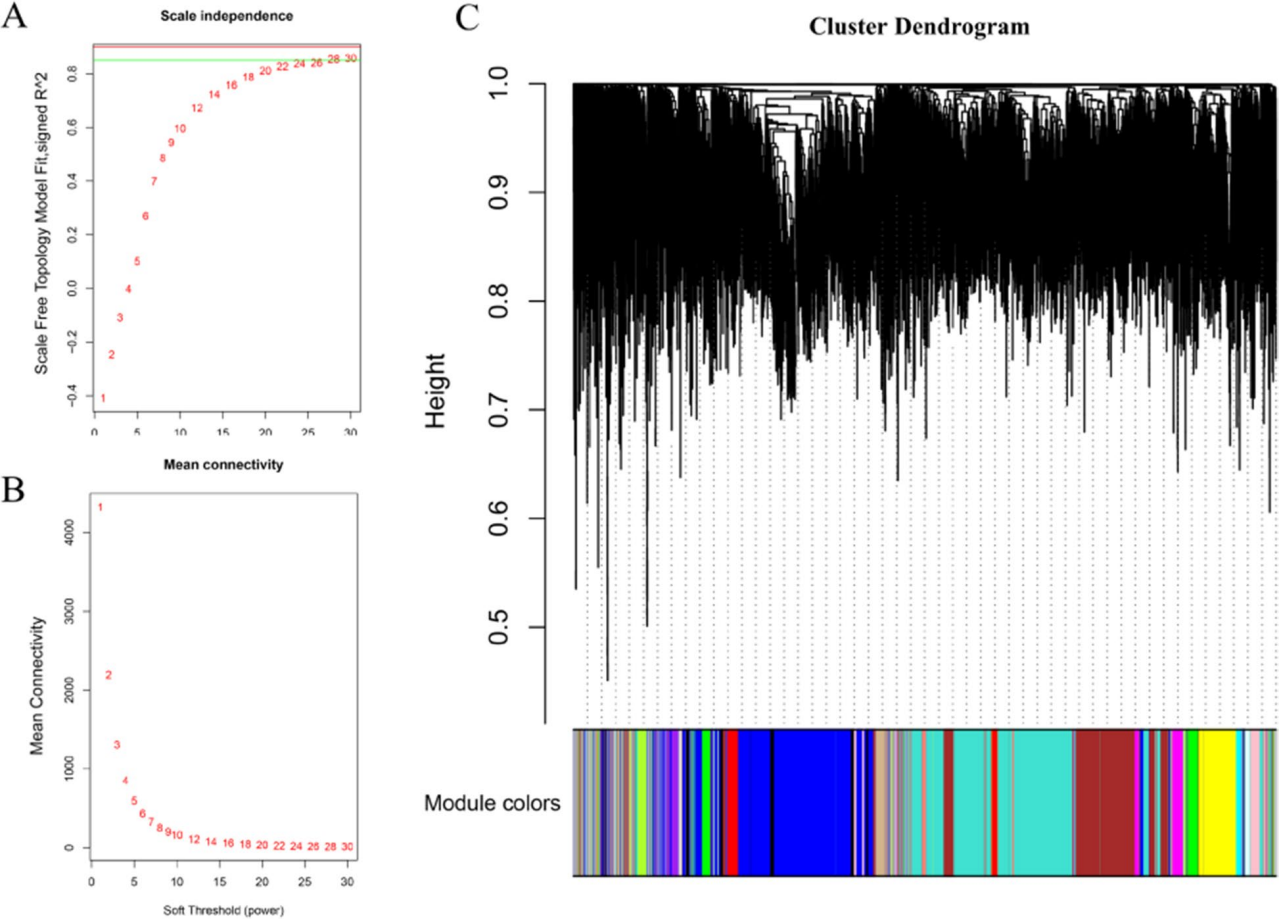
Weighted gene co-expression network construction and module identification​. **A** Scale-free index analysis of various soft threshold powers (β). **B** Average connectivity analysis of various soft threshold powers. **C** Clustering dendrogram of genes, with dissimilarity based on topological overlap, together with assigned module colors

The WGCNA network of 12,062 genes was performed using the WGCNA R software package. Through calculation, we find that when the soft threshold β = 28, the scale-free network was guaranteed, because the scale independence reached 0.9 (Fig. 1A) and had a relatively high-average connectivity (Fig. 1B). A total of 20 gene co-expression modules were identified, based on hierarchical clustering of the calculated dissimilarity (Fig. 1C). The largest module contains 2899 genes, the smallest module contains 33 genes, and the number of genes in the specific module was shown in Table S2. In addition, 928 uncorrelated genes were assigned into a gray module that was ignored in further analysis, and the information of genes in all module was shown in Table S3.

### FA composition trait-related modules in the breast muscle

**Fig. 2:**
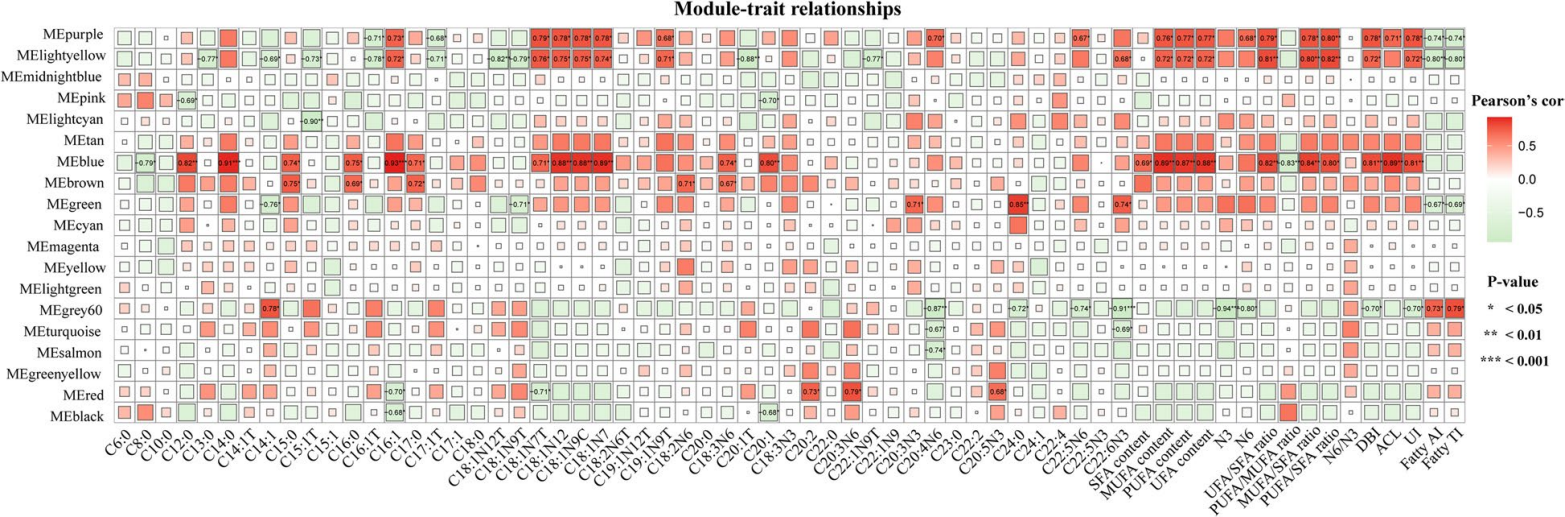
Relationships between modules and FA composition traits in the breast muscle of Gushi chicken. Each row in the table corresponds to a module and each column to a trait. Boxes contains Pearson correlation coefficients and their associated p values. Red color indicates a positive correlation between modules and traits, while green color indicates a negative correlation between modules and traits, only modules with a *p*-value < 0.05 are displayed

**Fig. 3:**
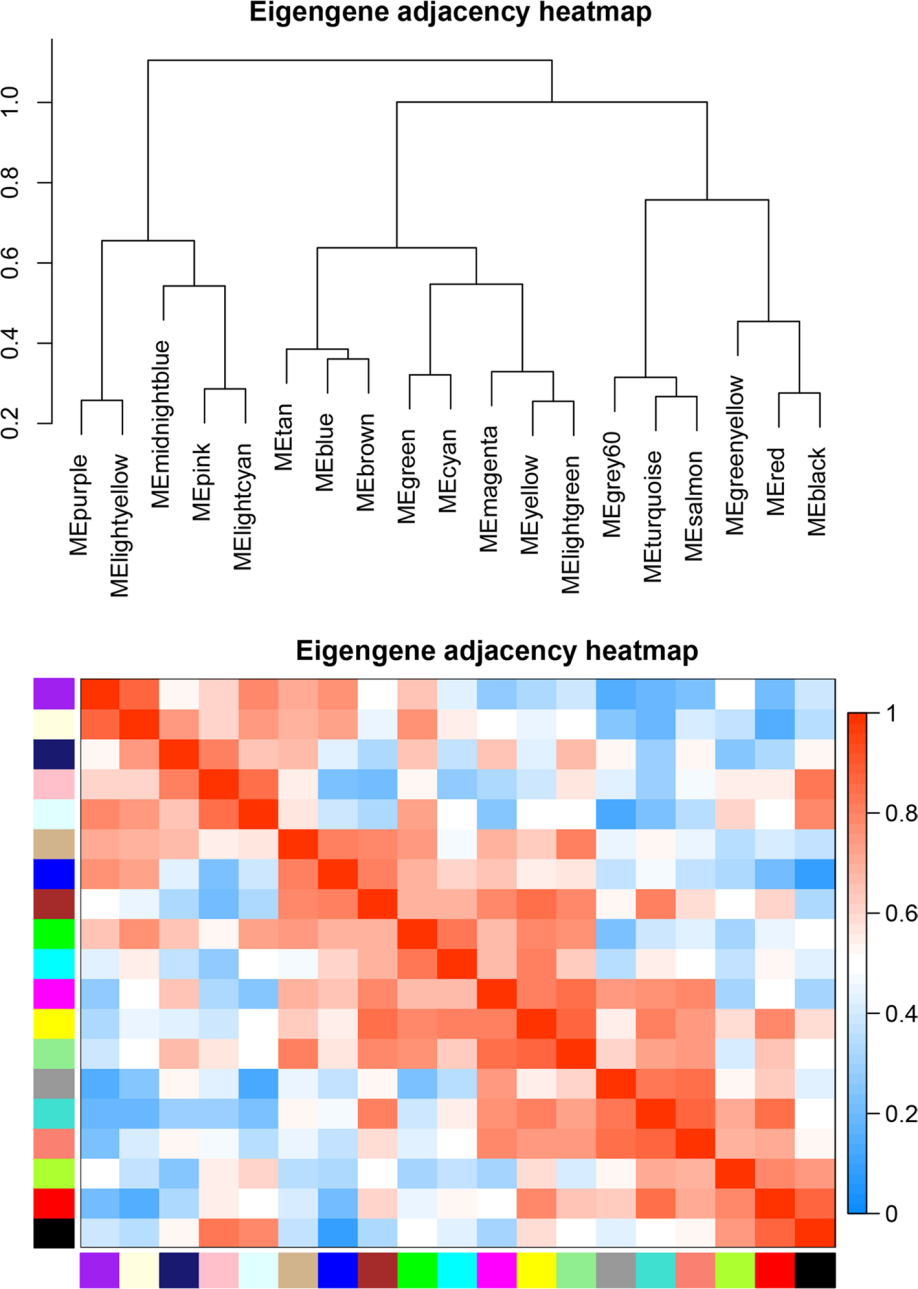
Relationships between modules. Branches of the dendrogram (the meta‐modules) group together eigengenes that are positively correlated. Each row and column in the heatmap corresponds to one module eigengene (labeled by color). Red represents high adjacency (positive correlation), while blue represents low adjacency (negative correlation). Squares of red color along the diagonal are the meta‐modules

In order to obtain specific modules that might be involved in FA composition in the breast muscle, this study evaluated the modular trait association between the 19 modules and 65 phenotypic traits. The results showed that six modules were highly correlated with fatty acid content and fatty acid metabolism traits (Fig. 2). Among the six modules, the purple and lightyellow modules were significant negative correlation to the C16:1 T, C17:1 T, Fatty.AI and Fatty,TI; the purple, lightyellow and blue modules were significant positive correlation to the C16:1, C18:1N7T, C18:1N12, C18:1N9C, C18:1N7, MUFA content, PUFA content, UFA content, UFA/SFA ratio, MUFA/SFA ratio, PUFA/SFA ratio, DBI, and UI in the breast muscle. In addition, the brown module was significantly positively correlated with C15:0, C16:0, C18:2N6 and C17:0; the green module was significantly positively correlated with C20:3N3, C24:0 and C22:6N3; the red module was significantly positively correlated with C20:2, C20:3N6 and C20:5N3. Through the cluster analysis of characteristic genes, the results show that the 19 modules could be divided into three clusters, and the modules had a high degree of interaction connectivity (Fig. 3). Among them, the purple and lightyellow modules belong to the same subclusters. In addition, the blue, brown and green modules belong to the same clusters, this suggests that these three modules had similar expression profiles. The red modules separately belong to another cluster. This suggests that genes in these six modules may play key roles during FA composition in the breast muscle of chickens.

### Enrichment analyses of module genes identified by weighted gene co-expression network analysis

**Fig. 4:**
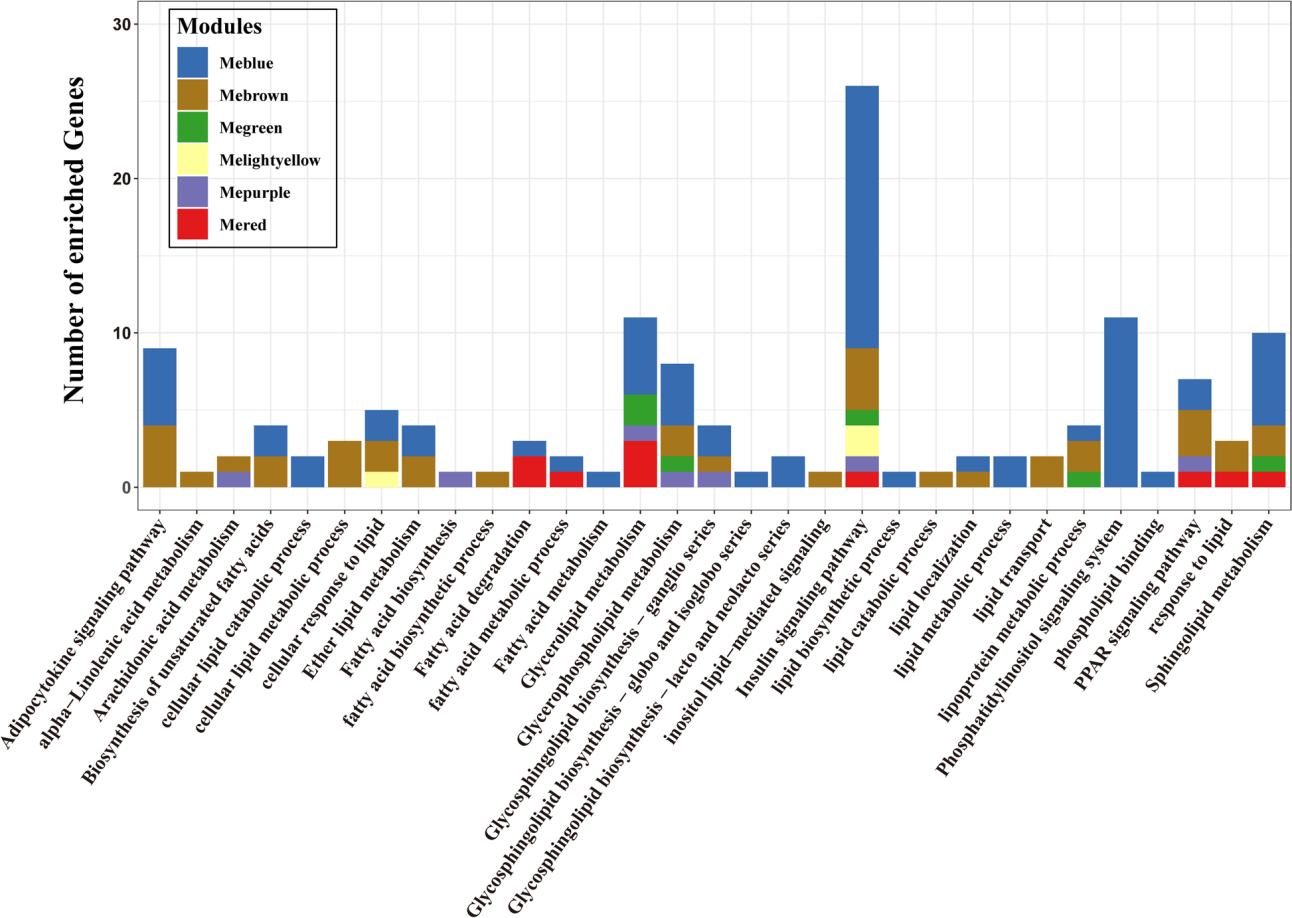
The lipid metabolism‐related pathways enriched in the six modules of interest. The abscissa indicates the enriched KEGG pathways. The ordinate indicates the numbers of enriched genes in each KEGG pathway

In order to explore the biological functions related to genes in six modules highly related to fatty acid composition, we conducted functional enrichment analysis. The results of GO enrichment analysis showed that the blue module was significantly enriched in the process of macromolecular catabolism, including protein catabolic process, cellular macromolecule catabolic process, etc. In the purple module, significant enrichment of ubiquitin-protein transferase activity related GO terms was found. In addition, the GO terms that were significantly enriched (corrected *P* < 0.05) were not found in the green, brown, lightyellow and red modules. The results of KEGG enrichment analysis showed that the blue module was significantly enriched in lysine degradation, citrate cycle (TCA cycle), cysteine and methionine metabolism, inositol phosphate metabolism and oxidative phosphorylation, etc. No significantly enriched signaling pathways were found in the other modules. Based on the above results, among the six modules, we identified 31 pathways associated with lipid metabolism or fat deposition (Fig. 4), including fatty acid metabolism, PPAR signaling pathway, fatty acid biosynthesis, biosynthesis of unsaturated fatty acids, etc. These results indicate that the genes in these modules may affect the fatty acid composition of chicken breast muscle through the above pathways or biological processes.

### The identification of hub genes and regulatory network construction

**Fig. 5:**
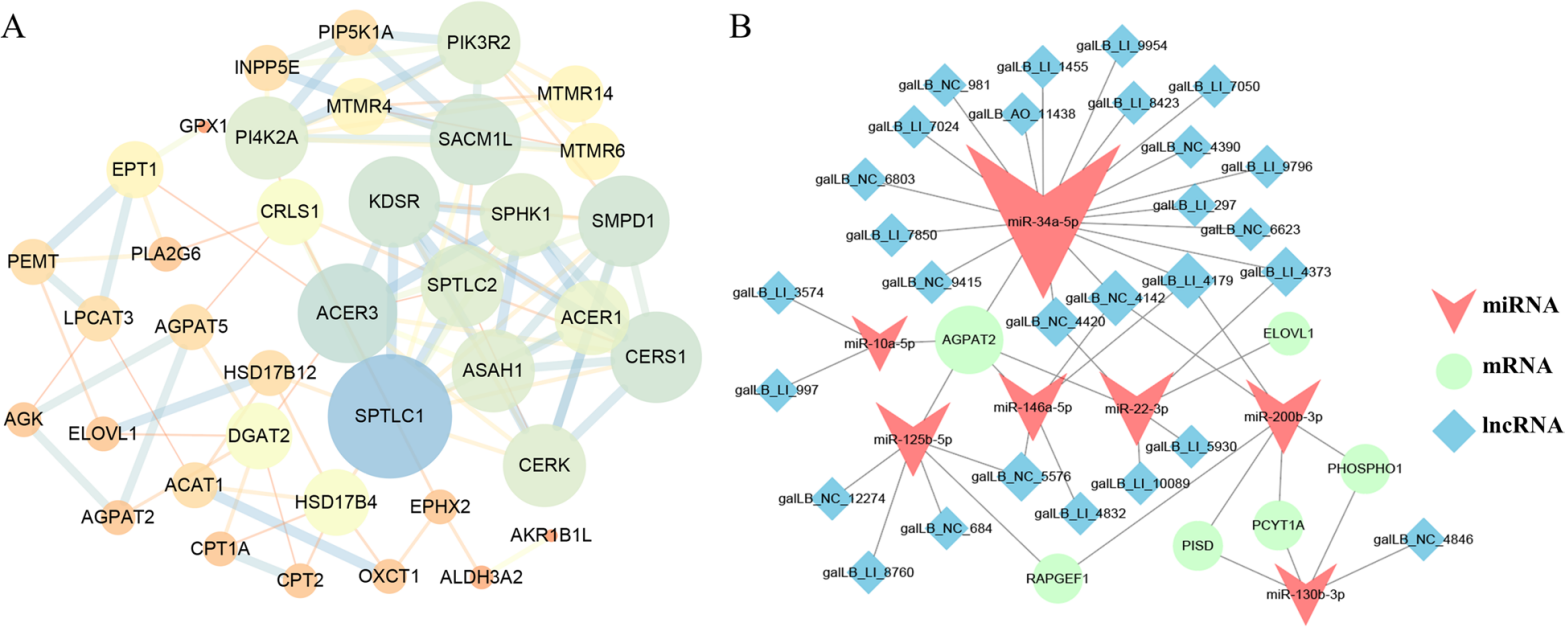
The interaction network and ceRNA regulatory network of hub genes related to lipid metabolism pathway. **A** The network was constructed using Cytoscape 3.7 software. The node size and edge number are proportional to degree and connection strength, respectively. **B** The lncRNA-miRNA-mRNA ceRNA network related to lipid metabolism pathway. Green represents mRNA, red represents miRNA, and blue represents lncRNA

Based on the criteria of |module membership (MM)|≥ 0.8 and |gene significance (GS)|≥ 0.2, we have identified a set of pivotal genes from different colored modules. Specifically, the blue, brown, green, light yellow, purple, and red modules yielded 72, 37, 6, 3, 7, and 10 pivotal genes, respectively. Through protein–protein interaction (PPI) network analysis, we have successfully identified a significantly enriched lipid metabolism pathway consisting of 38 genes (Fig. 5A). It is noteworthy that these nine genes are closely related to fatty acid metabolism, including *ACOT7*, *HSD17B4*, *ALDH3A2*, *CPT1A*, *ELOVL1*, *EPHX2*, *CPT2*, *GPX1*, and *HSD17B12*. Additionally, through an association analysis of transcriptomic data encompassing miRNA and lncRNA from breast muscle samples across four developmental stages, we have discovered a total of 51 crucial ceRNA regulatory networks, comprising 6 hub genes, 7 miRNA, and 28 lncRNA (Fig. 5B). Notably, these 6 hub genes (*RAPGEF1, AGPAT2, PISD, PCYT1A, PHOSPHO1, ELOVL1*) were closely associated with the synthesis and metabolism of fatty acids.

## Discussion

It is widely acknowledged that the types and levels of fatty acids are widely recognized for their impact on the flavor of meat [7]. In order to gain a comprehensive understanding of the hub genes and their post-transcriptional regulatory networks involved in fatty acid regulation in the breast muscles of Gushi chickens, we quantified the contents of 49 different fatty acids in Gushi chicken breast muscle at different developmental stages, the related data of 16 kinds of fatty acid metabolism were calculated. By combining WGCNA with previous transcriptomic data, we have identified six modules that are highly correlated with fatty acid phenotypic traits. Subsequently, we conducted enrichment analysis and identified 136 crucial genes that have a significant impact on fatty acid production by filtering hub genes associated with GS and MM. Lastly, we constructed 51 ceRNA regulatory networks centered around the hub genes by integrating miRNA and lncRNA data.

The determination of fatty acids in the breast muscle of Gushi chickens revealed that C16:0, C18:0, C18:1N12, C18:1N9C, C20:4N6, C18:1N7, C18:2N6, and C14:1T are the predominant fatty acids. Most of these predominant fatty acids align with findings from studies on other chicken breeds [23, 24]. Saturated fatty acids play a crucial role in muscle fatty acid composition, with C16:0 being the most abundant long-chain saturated fatty acid in Gushi chicken breast muscle. Long-chain fatty acids serve various important functions in the human body, such as providing energy, forming cell membranes, and participating in hormone synthesis, making them essential components of the diet [25–27]. Compared to other livestock and poultry, chicken meat exhibits higher levels of unsaturated fatty acids, which is consistent with the findings of this study [12]. Among the detected predominant fatty acids are C18:1N9C, C18:2N6, and C20:4N6. C18:2N6 is an essential fatty acid that cannot be synthesized by the human body and must be obtained through dietary intake [28]. It plays a critical role in maintaining the stability and function of cell membranes and it is involved in the synthesis of various bioactive substances, such as anti-inflammatory agents and cell signaling molecules [29–31]. These findings further highlight the primary reasons for the rich nutritional composition of Gushi chicken meat.

In order to further investigate the relationship between genes and fatty acid phenotypes, we conducted WGCNA analysis and defined a total of 19 modules. Among these modules, the blue, brown, green, light yellow, purple, and red modules showed the highest correlation with fatty acid phenotypes. The enrichment results of these six modules encompassed various signaling pathways related to lipid synthesis and metabolism, including fatty acid metabolism, glycerolipid metabolism, fatty acid biosynthesis, PPAR signaling pathway, cellular lipid degradation, and insulin signaling pathway. Through analysis of hub genes, we identified 136 genes that are associated with fatty acid phenotypes. PPI analysis revealed the involvement of multiple genes in fatty acid metabolism. For example, *ELOVL1* is involved in fatty acid chain elongation in the process of fatty acid synthesis [32]. It catalyzes the conversion of C18:00 fatty acyl-CoA to C20 and C22 fatty acids in the pathway of fatty acid synthesis [33]. Previous studies have shown that *ELOVL1* is upregulated during chicken preadipocyte differentiation, suggesting its important role in the accumulation of lipid droplets in chicken adipocytes [34]. Furthermore, *CPT1A* catalyzes the transfer reaction between long-chain fatty acids and carnitine, facilitating the transport of fatty acids into mitochondria for fatty acid oxidation and energy production [35, 36]. A study found that downregulation of *CPT1A* leads to reduced fatty acid oxidation and increased accumulation of intramuscular fat in chickens [37]. Additionally, genes such as *HSD17B4*, *ALDH3A2*, *EPHX2*, *CPT2*, *HSD17B12*, and *GPX1* are also involved in the oxidation process of fatty acids, although their specific roles may vary [38–42]. During the development of the Gushi chicken breast muscle, these genes may influence intramuscular fat content and fatty acid composition to varying degrees through their involvement in fatty acid oxidation. In addition, within the turquoise module, we identified the *DYNLL2* gene, which had previously been established in our earlier research as playing a pivotal role in the proliferation and differentiation of myoblasts in breast muscles [43]. In this study, the turquoise module housing this gene is not correlated with the traits related to breast muscle fatty acid composition. This observation serves to reinforce the accuracy of our study's findings, shedding light on an objective reality.

The main component of fat in muscles is phospholipids, which account for approximately 60% to 70% of muscle fat content [44]. Phospholipids primarily consist of components containing C16 and C18 fatty acids, which are major factors influencing the volatile flavor of meat [45, 46]. In this study, we identified nine key genes associated with the biosynthesis pathway of phospholipids. These genes are *PEMT*, *DGAT2*, *CRLS1*, *PLA2G6*, *AGK*, *LPCAT3*, *AGPAT5*, *EPT1*, and *AGPAT*. *AGPAT2* and *AGPAT5* are intermediate enzymes involved in the biosynthesis of phospholipids and triglycerides (TAGs) in the glycerol-3-phosphate pathway [47]. *AGPAT2* has been shown to play an important role in lipid metabolism in chickens [46]. *AGPAT5*, on the other hand, is considered a potential genetic marker for excellent pork quality [48]. In a study conducted on cattle, *AGPAT5* was found to be significantly correlated with pre-slaughter weight, carcass weight, and meat weight [49]. Additionally, *DGAT2* is a key gene involved in regulating the synthesis of triglycerides (TGs), as it facilitates the storage of excess fatty acids in lipid droplets [47]. In other studies, *DGAT2* has been suggested to potentially regulate the deposition of intramuscular fat (IMF) in chicken meat [50]. These genes may regulate lipid deposition in the pectoralis muscle of Gushi chickens through the biosynthesis of phospholipids.

In this study, through the combined analysis with previous transcriptome data, a transcriptional post-regulatory network of some hub genes was discovered. Specifically, miR-34a-5p is likely regulated by multiple lncRNAs, thereby affecting the expression of *AGPAT2*. It is known that miR-34a-5p increases intracellular triglyceride and total cholesterol levels in chicken liver by inhibiting the *ACSL1* gene [51]. In intramuscular fat, miR-34a-5p may regulate fat deposition through different pathways. Additionally, there is a potential regulatory network between miR-130b-3p and *PISD*, *PCYT1A*, and *PHOSPHO1*, which is regulated by galLB_NC_4846. miR-130b-3p is known to negatively regulate the accumulation of lipid droplets in goat intramuscular preadipocytes [52]. *PISD* and *PCYT1A* are associated with fatty acid synthesis and metabolism [47]. Furthermore, there is a potential targeting relationship between *ELOVL1* and miR-22-3p. miR-22-3p is known to target *ELOVL6* to regulate lipid synthesis in chicken liver [53]. Therefore, miR-22-3p may affect fat deposition in muscle by regulating *ELOVL1*. These findings suggest that post-transcriptional regulatory mechanisms, especially miRNAs and lncRNAs, play an important regulatory role in intramuscular fat deposition.

## Conclusions

In conclusion, based on the data from fatty acids and transcriptomics in the breast muscles of Gushi chickens, we have identified signaling pathways highly associated with fatty acid content and composition. Through an analysis focused on hub genes and post-transcriptional regulatory mechanisms, we have defined a number of key genes and their interconnected ceRNA regulatory network. The study of these key genes may provide us with new insights into the formation of the flavor characteristics in the meat of Gushi chickens.

## Materials and methods

### Sample collection

In this study, Gushi chicken, a Chinese local chicken breed was used as the experimental animal from the Animal Center of Henan Agricultural University. Female chickens were raised in ladder cage under the same environment and standard feeding conditions. The feeding method was the same as our previous study [54]. In this study, three healthy chickens were randomly selected at 14, 22, and 30 weeks of age. Therefore, nine chickens were used in this study. Chickens were anesthetized by intravenous injection of sodium pentobarbital (40 mg/kg) at a concentration of 0.2% in the wing vein. Under deep anesthesia, these individuals were euthanized by intravenous KCL (1 ~ 2 mg/kg). Then their breast muscles tissues were collected, immediately frozen in liquid nitrogen and stored at − 80℃.

### Collection of phenotypic data with meat traits

To obtain meat quality trait phenotypic data, we analyzed the different fatty acid composition contents of Gushi chicken breast muscles tissues at three developmental stages: 14, 22, and 30 weeks of age. The content of fatty acids in breast muscle tissue was performed by Trace 1310-ISQ 7000 gas chromatography-mass spectrometry (GC–MS) (Thermo Fisher Scientific, Shanghai, China), in accordance with the PRC National Standard “Meat and meat products—determination of fatty acids (GB/T 9695.2–2008).” In plain terms, we assessed the 49 kinds of fatty acids content in Gushi chicken breast muscle tissue and 16 kinds of fatty acid metabolism traits. The fatty acids content was expressed as the percentage of the breast muscle by fresh weight (μg/g).

### mRNA expression data

The processing and analysis of mRNA expression data from the previously constructed Gushi chicken breast muscle transcriptome expression profile [55], with the sample accession of mRNA expression data of PRJNA516810. A total of 16,682 expressed genes were identified in Gushi chicken breast muscle tissue. Using the R language for data filtering, removing all genes that have a count of less than say 10 in more than 90% of the samples, the code was "gene_filter <—readcount[apply(readcount, 1, function(x) sum(x > 10) > (0.9 * ncol(readcount))),]". The resulting set of genes, composed of 12,062 genes post-filtering, was normalized using the CPM function from the edgeR package and then used in the subsequent WGCNA analysis.

### Weighted gene co-expression network analysis

Weighted gene co-expression network was constructed for 12,062 genes using the WGCNA R software package [15]. Firstly, the Pearson correlation coefficient between each gene pair is calculated by using CPM value of selected genes, which is expressed as the connection strength between genes. The appropriate β value was selected through he R function PickSoftThreshold. Subsequently, we converted the adjacency matrix into the topological overlap matrix (TOM) to minimize the effects of noise and false positives by considering the (indirect) calculation method of intermediate nodes. Next, hierarchical clustering was used to identify modules and merged similar modules when each module contained at least 30 genes (minModuleSize = 30) and the threshold of merging similar modules was 0.25 (mergeCutHeight = 0.25).

### Construct module-trait relationships

In order to identify the modules significantly related to FA composition, we performed principal component analysis on the gene expression matrix in each module, and used the module eigengene (ME) to represent the first principal component (PC1) of each module. Then, the modular trait association between MEs and phenotypic traits was evaluated by Pearson correlation coefficient. The correlation between each module and each trait was also indicated by the heatmap.

### Functional enrichment analysis of genes in modules of interest

In order to explore the biological function of module genes related to fatty acid composition in breast muscle of Gushi chicken, R software package clusterProfile 4.0 was used for enrichment analyses of Gene Ontology (GO), Kyoto Encyclopedia of Genes and Genomes (KEGG) [56]. The *P*-value < 0.05 was used as the enrichment cut-off criterion, and the visualization was performed using ggplot2.

### Hub genes analysis

Under the weighted co-expression network, highly connected genes are called hub genes, which may play an important role in the biological regulatory mechanisms of the living organism. In this network, hub gene was determined by the correlation between gene expression profile and module membership (MM) and the correlation between gene and external traits (GS). Thus, the intramodular hub genes were chosen by |GS|> 0.2 and |MM|> 0.8 with a threshold of *P*-value < 0.05.

The expression of genes was controlled by various factors, such as the regulation of non-coding RNA, including miRNA, lncRNA, etc. Therefore, we used the identified differential miRNA and differential lncRNA data at 14, 22, 30 weeks from the same RNA sample transcriptome and small RNA library collected from the breast muscle tissues of Gushi chicken [54, 57]. The data were used to construct the lncRNA-miRNA-mRNA regulatory network by association analysis of hub gene, which is highly correlated with the fatty acid composition of the breast muscles. The Cytoscape 3.7 software was used for the visualization of the network [58].

### Supplementary Information

**Additional file 1:** **Table S1. **The phenotypic value associated with FA composition in Gushi chicken breast muscle at different developmental stages.

**Additional file 2:** **Table S2. **The number of known genes in the 20 modules.

**Additional file 3:** T**able S3.**  The information of known genes in the 19 modules.

## Data Availability

The authors declare that the data supporting the findings of this study are available within the article and its supplementary information files. All the raw sequences have been deposited in the NCBI database Sequence Read Archive with the BioProject number PRJNA516810 (mRNA and lncRNA) and PRJNA516961 (miRNA).
