# Supplementary material for: Weighted gene co-expression network analysis identified hub genes critical to fatty acid composition in Gushi chicken breast muscle
Source: BMC Genomics. 2023 Oct 7;24:594. doi: 10.1186/s12864-023-09685-8 (PMC10559426; doi:10.1186/s12864-023-09685-8)
Supplement: Supplementary file 1 — Additional file 1: Table S1. The phenotypic value associated with FA composition in Gushi chicken breast muscle at different developmental stages. [file 12864_2023_9685_MOESM1_ESM.docx]

**Table S1. The phenotypic value associated with FA composition in Gushi chicken breast muscle at different developmental stages​.**

| **Items** | **W14-1** | **W14-2** | **W14-3** | **W22-1** | **W22-2** | **W22-3** | **W30-1** | **W30-2** | **W30-3** |
| --- | --- | --- | --- | --- | --- | --- | --- | --- | --- |
| Caproate (C6:0) | 1.58 | 2.29 | 2.05 | 2.35 | 2.49 | 2.04 | 1.65 | 2.01 | 2.29 |
| Caprylate (C8:0) | 1.53 | 1.68 | 1.56 | 1.66 | 2.01 | 1.75 | 1.29 | 1.57 | 1.88 |
| Caprate (C10:0) | 0.32 | 0.30 | 0.36 | 0.32 | 0.42 | 0.31 | 0.29 | 0.28 | 0.35 |
| Laurate (C12:0) | 0.70 | 0.90 | 0.86 | 0.69 | 0.68 | 0.78 | 1.02 | 0.85 | 0.78 |
| Tridecanoate (C13:0) | 1.63 | 3.64 | 2.57 | 2.50 | 2.56 | 1.82 | 1.76 | 2.07 | 1.99 |
| Myristate (C14:0) | 9.48 | 10.71 | 9.52 | 8.20 | 8.40 | 8.52 | 17.14 | 12.13 | 8.64 |
| Myristelaidate (C14:1T) | 71.90 | 107.40 | 55.01 | 75.71 | 43.20 | 77.78 | 63.91 | 66.35 | 76.83 |
| Myristoleate (C14:1 | 21.29 | 22.22 | 20.47 | 23.50 | 13.27 | 24.65 | 12.53 | 12.66 | 17.44 |
| Pentadecanoate (C15:0) | 1.40 | 1.89 | 1.56 | 1.28 | 1.31 | 1.32 | 1.95 | 1.66 | 1.46 |
| 10-Transpentadecenoate (C15:1T) | 9.61 | 11.41 | 11.16 | 10.36 | 10.14 | 11.28 | 9.71 | 9.21 | 9.72 |
| 10-Pentadecenoate (C15:1) | 8.21 | 10.43 | 8.83 | 9.51 | 8.60 | 11.90 | 8.46 | 7.70 | 12.48 |
| Palmitate (C16:0) | 933.54 | 1196.28 | 1060.73 | 877.57 | 1005.50 | 940.58 | 1217.77 | 956.98 | 915.92 |
| Palmitelaidate (C16:1T) | 23.46 | 27.48 | 23.86 | 23.77 | 22.14 | 26.05 | 21.06 | 20.93 | 22.96 |
| Palmitoleate (C16:1) | 19.13 | 21.45 | 19.12 | 19.41 | 18.54 | 20.70 | 58.05 | 26.02 | 19.25 |
| Heptadecanoate (C17:0) | 3.17 | 3.87 | 2.93 | 2.54 | 2.83 | 2.70 | 4.01 | 3.11 | 2.85 |
| 10-Transsheptadecenoate (C17:1T) | 10.41 | 13.00 | 10.37 | 11.12 | 10.74 | 12.09 | 9.63 | 9.61 | 10.45 |
| 10-Heptadecenoate (C17:1) | 12.21 | 19.37 | 20.50 | 11.88 | 18.75 | 14.20 | 18.76 | 11.95 | 14.47 |
| Stearate (C18:0) | 542.35 | 693.34 | 617.63 | 528.06 | 623.74 | 547.84 | 658.30 | 545.59 | 557.89 |
| Petroselaidate (C18:1N12T) | 7.63 | 9.27 | 8.07 | 8.18 | 8.06 | 8.15 | 7.33 | 6.97 | 7.98 |
| Elaidate (C18:1N9T) | 4.26 | 4.77 | 4.04 | 4.16 | 4.03 | 4.22 | 3.67 | 3.49 | 4.00 |
| Transvaccenate (C18:1N7T) | 29.99 | 29.54 | 24.94 | 26.81 | 26.01 | 29.93 | 59.08 | 33.36 | 41.04 |
| Petroselinate (C18:1N12) | 183.06 | 159.85 | 98.82 | 87.82 | 134.35 | 103.07 | 638.45 | 228.75 | 131.10 |
| Oleate (C18:1N9C) | 175.62 | 153.36 | 94.84 | 84.29 | 128.91 | 98.92 | 612.27 | 219.42 | 125.80 |
| Vaccenate (C18:1N7) | 161.68 | 137.58 | 79.57 | 61.99 | 129.74 | 74.31 | 616.18 | 201.44 | 82.51 |
| Linoelaidate (C18:2N6T) | 1.74 | 1.92 | 1.83 | 1.82 | 1.71 | 1.84 | 1.98 | 1.47 | 1.78 |
| 7-Transnonadecenoate (C19:1N12T) | 6.68 | 6.91 | 5.70 | 5.30 | 5.84 | 5.79 | 7.17 | 5.34 | 6.29 |
| 10-Transnonadecenoate (C19:1N9T) | 3.08 | 2.54 | 1.28 | 2.35 | 1.56 | 1.91 | 4.64 | 3.23 | 2.51 |
| Linoleate (C18:2N6) | 147.13 | 131.31 | 68.96 | 79.26 | 84.55 | 62.28 | 154.39 | 139.11 | 59.27 |
| Arachidate (C20:0) | 4.15 | 5.30 | 4.57 | 3.88 | 4.70 | 4.30 | 4.91 | 4.06 | 4.61 |
| Gamma Linolenate (C18:3N6) | 1.48 | 1.56 | 0.77 | 0.72 | 0.81 | 0.92 | 2.14 | 1.28 | 0.79 |
| Trans 11-Eicosenoate (C20:1T) | 7.94 | 9.95 | 9.00 | 8.38 | 8.32 | 8.39 | 7.70 | 7.60 | 7.96 |
| 11-Eicosenoate (C20:1 | 9.24 | 11.30 | 10.58 | 8.53 | 9.01 | 9.28 | 12.48 | 8.25 | 8.83 |
| Alpha Linolenate (C18:3N3) | 3.68 | 2.42 | 0.86 | 0.81 | 0.98 | 0.66 | 4.17 | 3.16 | 1.16 |
| 11-14 Eicosadienoate (C20:2) | 4.64 | 5.53 | 4.04 | 3.62 | 4.13 | 2.66 | 3.35 | 3.58 | 3.08 |
| Behenate (C22:0) | 0.88 | 0.95 | 0.79 | 0.40 | 0.63 | 0.63 | 1.01 | 0.60 | 0.96 |
| Homogamma Linolenate (C20:3N6 | 9.46 | 10.92 | 7.05 | 7.74 | 7.25 | 7.63 | 6.36 | 7.08 | 7.29 |
| Brassidate (C22:1N9T) | 6.88 | 8.54 | 8.67 | 8.08 | 8.02 | 7.79 | 7.24 | 7.34 | 7.93 |
| Erucate (C22:1N9) | 6.17 | 7.28 | 8.89 | 6.64 | 7.54 | 6.25 | 6.59 | 6.89 | 6.42 |
| 11-14-17 Eicosatrienoate (C20:3N3) | 0.60 | 0.53 | 0.63 | 0.63 | 0.68 | 0.38 | 0.65 | 0.77 | 0.61 |
| Arachidonate (C20:4N6) | 109.00 | 148.39 | 125.22 | 83.32 | 163.35 | 103.19 | 228.22 | 158.00 | 189.66 |
| Tricosanoate (C23:0) | 0.28 | 0.34 | 0.27 | NA | 0.32 | 0.25 | 0.35 | 0.21 | 0.21 |
| Docosadienoate (C22:2) | 1.59 | 1.80 | 2.06 | 1.45 | 2.24 | 1.45 | 1.36 | 1.47 | 1.32 |
| Eicosapentaenoate (C20:5N3) | 1.95 | 2.29 | 1.32 | 1.07 | 1.64 | 1.08 | 1.20 | 1.35 | 1.26 |
| Lignocerate (C24:0) | 0.30 | 0.33 | 0.36 | 0.33 | 0.37 | 0.28 | 0.39 | 0.48 | 0.42 |
| Nervonoate (C24:1) | 5.60 | 6.06 | 6.33 | 5.88 | 6.49 | 6.50 | 6.02 | 5.83 | 6.37 |
| Docosatetraenoate (C22:4) | 27.48 | 28.08 | 21.63 | 20.43 | 39.23 | 25.53 | 26.39 | 28.38 | 30.75 |
| Docosapentaenoate (C22:5N6) | 10.72 | 11.74 | 13.06 | 8.07 | 16.79 | 9.91 | 19.09 | 14.88 | 13.21 |
| Docosapentaenoate (C22:5N3) | 13.79 | 18.54 | 15.19 | 9.21 | 18.57 | 12.22 | 14.85 | 12.37 | 15.89 |
| Docosahexaenoate (C22:6N3) | 17.50 | 19.99 | 20.83 | 11.40 | 24.59 | 14.35 | 38.79 | 34.66 | 34.05 |
| SFA content | 1501.30 | 1921.83 | 1705.75 | 1429.80 | 1655.96 | 1513.12 | 1911.84 | 1531.59 | 1500.26 |
| MUFA content | 784.06 | 779.71 | 530.07 | 503.66 | 623.26 | 563.16 | 2190.93 | 902.35 | 622.35 |
| PUFA content | 1134.81 | 1164.74 | 813.51 | 733.21 | 989.78 | 807.26 | 2693.87 | 1309.90 | 982.49 |
| UFA content | 1918.86 | 1944.45 | 1343.58 | 1236.87 | 1613.04 | 1370.43 | 4884.79 | 2212.25 | 1604.84 |
| N3 | 37.51 | 43.78 | 38.83 | 23.12 | 46.45 | 28.68 | 59.66 | 52.31 | 52.97 |
| N6 | 279.53 | 305.84 | 216.88 | 180.93 | 274.47 | 185.77 | 412.18 | 321.82 | 272.01 |
| UFA:SFA ratio | 1.28 | 1.01 | 0.79 | 0.87 | 0.97 | 0.91 | 2.56 | 1.44 | 1.07 |
| PUFA:MUFA ratio | 1.45 | 1.49 | 1.53 | 1.46 | 1.59 | 1.43 | 1.23 | 1.45 | 1.58 |
| MUFA:SFA ratio | 0.52 | 0.41 | 0.31 | 0.35 | 0.38 | 0.37 | 1.15 | 0.59 | 0.41 |
| PUFA:SFA ratio | 0.76 | 0.61 | 0.48 | 0.51 | 0.60 | 0.53 | 1.41 | 0.86 | 0.65 |
| N6:N3 | 7.45 | 6.99 | 5.59 | 7.82 | 5.91 | 6.48 | 6.91 | 6.15 | 5.14 |
| DBI | 1923.07 | 2095.83 | 1571.95 | 1280.81 | 1980.54 | 1445.41 | 3979.94 | 2326.95 | 2020.59 |
| ACL | 456.35 | 531.54 | 433.77 | 370.52 | 460.28 | 398.24 | 808.35 | 494.92 | 431.38 |
| UI | 3846.14 | 4191.66 | 3143.90 | 2561.62 | 3961.09 | 2890.83 | 7959.87 | 4653.89 | 4041.18 |
| Fatty AI | 0.88 | 1.10 | 1.40 | 1.29 | 1.10 | 1.25 | 0.48 | 0.79 | 1.00 |
| Fatty TI | 2.30 | 2.82 | 3.44 | 3.43 | 2.78 | 3.25 | 1.28 | 1.97 | 2.45 |

Abbreviation: W14, sample 1 of 14 weeks; W22, sample 1 of 22 weeks; W30, sample 1 of 30 weeks; SFA, saturated fatty acid; MUFA, monounsaturated fatty acid; PUFA, polyunsaturated fatty acid; UFA, unsaturated fatty acids; N3, n-3 polyunsaturated fatty acids; N6, n-6 polyunsaturated fatty acid; DBI, double bond index; ACL, average carbon length; UI, Unsaturation Index; Fatty AI, fatty acid arteriosclerosis index; Fatty TI, fatty acid thrombogenicity index.
