# Supplementary material for: Weighted gene co-expression network analysis identified hub genes critical to fatty acid composition in Gushi chicken breast muscle
Source: BMC Genomics. 2023 Oct 7;24:594. doi: 10.1186/s12864-023-09685-8 (PMC10559426; doi:10.1186/s12864-023-09685-8)
Supplement: Supplementary file 2 — Additional file 2: Table S2. The number of known genes in the 20 modules. [file 12864_2023_9685_MOESM2_ESM.docx]

**Table S2. The number of known genes in the 20 modules.**

| Module | Number of genes | Module | Number of genes |
| --- | --- | --- | --- |
| ME black | 314 | ME lightyellow | 33 |
| ME blue | 2852 | ME magenta | 287 |
| ME brown | 1796 | ME midnightblue | 100 |
| ME cyan | 100 | ME pink | 297 |
| ME green | 421 | ME purple | 229 |
| ME greenyellow | 195 | ME red | 353 |
| ME grey | 928 | ME salmon | 120 |
| ME grey60 | 47 | ME tan | 194 |
| ME lightcyan | 97 | ME turquoise | 2899 |
| ME lightgreen | 41 | ME yellow | 759 |
